# Tomographic Study of the Malformation Complex in Correlation With the Genotype in Patients With Robinow Syndrome: Review Article

**DOI:** 10.1177/2324709620911771

**Published:** 2020-03-14

**Authors:** Ali Al Kaissi, Vladimir Kenis, Mohammad Shboul, Franz Grill, Rudolf Ganger, Susanne Gerit Kircher

**Affiliations:** 1 Hanusch Hospital, Vienna, Austria; 2 Orthopedic Hospital of Speising, Vienna, Austria; 3 Pediatric Orthopedic Institute n.a. H. Turner, Saint-Petersburg, Russia; 4 Jordan University of Science and Technology, Irbid, Jordan; 5 Medical University of Vienna, Vienna, Austria

**Keywords:** Robinow syndrome, autosomal recessive and dominant types, 3D, reconstruction CT scan, genotype

## Abstract

We aimed to understand the etiology behind the abnormal craniofacial contour and other clinical presentations in a number of children with Robinow syndrome. Seven children with Robinow syndrome were enrolled in this study (autosomal recessive caused by homozygous mutations in the *ROR2* gene on chromosome 9q22, and the autosomal dominant caused by heterozygous mutation in the WNT5A gene on chromosome 3p14). In the autosomal recessive (AR) group, the main clinical presentations were intellectual, disability, poor schooling achievement, episodes of headache/migraine, and poor fine motor coordinative skills, in addition to massive restrictions of the spine biomechanics causing effectively the development of kyposcoliosis and frequent bouts of respiratory infections. Three-dimensional reconstruction computed tomography scan revealed early closure of the metopic and the squamosal sutures of skull bones. Massive spinal malsegmentation and unsegmented spinal bar were noted in the AR group. In addition to severe mesomelia and camptodactyly, in the autosomal dominant (AD) group, no craniosynostosis but few Wormian bones and the spine showed limited malsegemetation, and no mesomelia or camptodactyly have been noted. We wish to stress that little information is available in the literature regarding the exact pathology of the cranial bones, axial, and appendicular malformations in correlation with the variable clinical presentations in patients with the 2 types of Robinow syndrome.

## Introduction

This syndrome was first described by Robinow.^1^ The facial features are described to resemble those of a fetus, with a bulging frontal area of the skull, increased orbital distance, a wide mouth, and a small nose. Apparent gingival hyperplasia and mesomelic limb shortening are infrequent abnormalities. Additional abnormalities include small penis in males, hydronephrosis, orofacial clefts, and spine malsegmentation. Additional abnormalities include brachydactyly with a small hand for age, abnormal metacarpophalangeal pattern profile with the distal phalanges of the third and fourth digits being less affected than the second and fifth, and with the first proximal phalanx relatively less shortened than the first metacarpal, and usually short fifth middle phalanx, with or without clinodactyly.^2^

There were several synonyms for Robinow syndrome (RS; such as fetal face; Robinow-Silverman; mesomelic dysplasia, type Robinow; costovertebral segmentation defect with mesomelia [autosomal recessive (AR)]; COVESDEM syndrome).^2-4^ RS can be inherited as autosomal recessive (ARRS; OMIM 268310) and autosomal dominant (ADRS; OMIM 180700).^1,5,6^

Robinow syndrome is a rare heterogeneous heritable disorders caused by mutations in genes, for example, *WNT5A* (MIM 164975), *ROR2* (MIM 602337), *FZ2* (MIM 258315), *DVL* (*DVL1*; MIM 601365, *DVL3*; MIM: 601368), and *NXN* (MIM 612895).^7^ These genes encode for components of the Wnt signaling, which controls convergent extension: a polarized cell migration that narrows and extends the body axis during vertebrate gastrulation as well as limb development and skeletal morphogenesis and regulates cell polarity, motility, survival, proliferation, and differentiation.^8-11^

Autosomal recessive Robinow syndrome is typically the more severe form of RS caused by biallelic loss-of-function mutations in *ROR2, WNT5A*, and *NXN*.^5,7,12,13^ Heterozygous mutations in the *ROR2* gene is also associated with a distinct syndrome, the autosomal dominant brachydactyly type B1 (BDB1, OMIM 113000); however, a small phenotypic overlap has to be noted.^14,15^ Homozygous mutations in *ROR2* and *WNT5A* were found to impair the WNT5A/ROR2 signal pathway resulting in skeletal abnormalities and other features characteristic of RS.^8,16-18^ RS phenotype has been discussed comprehensively in zebrafish, *Xenopus*, and mouse models. Knockout of *Ror2* and *Wnt5a* exhibited phenotypes similar to those found in RS patients.^16,19-21^ Hypoplastic phalanges in *Ror2*^−/−^ embryos were also shown as compared with the wild type, reflecting the mild brachydactyly seen in some ARRS patients.^22^ Wnt5a was also found to be necessary for kidney development in both mouse and zebrafish phenocopied to RS patients.^5,15,16,23,24^ Moreover, the *NXN* gene encodes nucleoredoxin, a regulator of the Wnt/PCP pathway through binding with DVL. *Nxn*^−/−^ mouse models showed abnormal differentiation of osteoblastic cells, craniofacial abnormalities with a shortened nose, and cleft palate partially recapitulating the RS subjects’ phenotype.^7,25^

On the other hand, the milder autosomal dominant RS has been found to be caused by heterozygous mutations in *WNT5A, DVL1*, and *DVL3*. DVL is a highly conserved central mediator of Wnt signaling including the noncanonical Wnt/PCP pathway via interaction with ROR2 or by binding to the cytoplasmic frizzled family members and transducing the Wnt signal to downstream effectors.^26,27^ All reported mutations in *DVL1* and *DVL3* led to perturbation of Wnt pathway and showed similar features associated with ADRS.^7,28-30^ Mice and *Xenopus* models for *Dvl* paralogs showed short stature, axial skeleton defects, and craniofacial malformations.^31,32^ Recently identified variants in *FZD2* (a Wnt receptor) associated ADRS and autosomal-dominant omodysplasia, an RS-like phenotype (OMOD2; OMIM 16475).^7,32,33^ *Fzd2*^−/−^ mice also displayed lethal phenotype compatible with RS patients phenotype.^34^ Furthermore, mutations in the receptor-like tyrosine kinase gene (*RYK*, MIM 600524) might also cause RS, but no mutations yet were identified in RS-affected patients. Ryk is essential for Wnt5a/PCP regulation in multiple developmental processes. Ryk together with the Ror2 were found to regulate the Wnt5a (Wnt/PCP) signaling via interacting with Vangl2.^12,35^ These findings were supported in *Ryk*-deficient mice that showed typical PCP defects similar to *Wnt5a*- and *Ror2*-null mice.^35-37^ Therefore, mutations in human *RYK* might cause RS and brachydactyly and provide new insight into the etiology of these diseases.

Collectively, the pathogenesis of RS because of mutations in the RS-associated genes appears to be a result of perturbation of Wnt/PCP signaling. Locus heterogeneity characterizes a variety of skeletal dysplasia due to interacting or overlapping signaling pathways. The contribution of distinct genes from the same pathway may explain locus heterogeneity in RS.

## Material and Methods

Ethical approval to report this case series was obtained from the Ethics Committee of the Turner Scientific Research Institute, No.3/2016, Pediatric Orthopedic Institute n.a. H. Turner, Department of Foot and Ankle Surgery, Neuroorthopaedics and Systemic Disorders, Parkovaya, Pushkin Saint-Petersburg, Russia. Written informed consent was obtained from the patients’ guardians for their anonymized information to be published in this article. This study was conducted based on clinical and radiographic phenotypic interpretations of a group of children and was carried out between January 1, 2009, and March 2018. All patients showed the classical abnormalities of RS. Nevertheless, we noticed different clinical presentations between patients with AD and AR types of RS. The study included 7 unrelated patients (4 girls and 3 boys) who have been diagnosed and assessed by the first author. All presented clinically with the clinical and the radiological phenotypic characterizations of RS. The genotype confirmed the diagnosis of Robinow, and we further subdivided our group of patients into the AR form (3 patients underwent genetic tests; 2 of them showed a loss-of-function mutation in the *ROR2* gene encoding a putative WNT5A receptor) and the AD form (4 patients; 2 of them showed mutations of *WNT5A* encoding a protein in the WNT signaling pathway). Conventional radiographic study via skeletal survey evaluation showed unclarified differences between the ARRS and the ADRS groups. The incentive to drive us to perform comprehensive tomographic analysis in both patient groups is to further understand the diversity in their complaints and the reasons behind the unusual clinical presentations.

## Results

### Group I: Autosomal Recessive Pattern of Inheritance (RRS) Caused by Homozygous Mutations in the *ROR2* Gene on Chromosome 9q22

**Figure 1.**
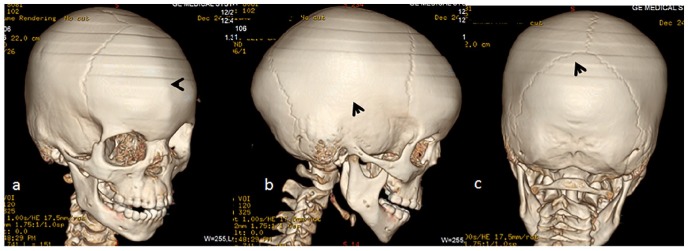
Craniosynostosis. (a) Three-dimensional reconstruction computed tomography (CT) scan of the skull in a 7-year-old girl with autosomal recessive Robinow syndrome (ARSS) showed early closure of the metopic suture (arrowhead) associated with profound bossing of the frontal area (more marked at the glabella). Note the normalcy of the coronals and acute depression of the nasofrontal angle and dysplastic supraorbital ridge. (b) Three-dimensional reconstruction CT scan in a patient with ARSS showed early closure of the squamosal sutures (arrowhead). (c) Three-dimensional reconstruction CT scan in a patient with ARSS showed normal lambdoid sutures with no trace of Wormian bones.

**Figure 2.**
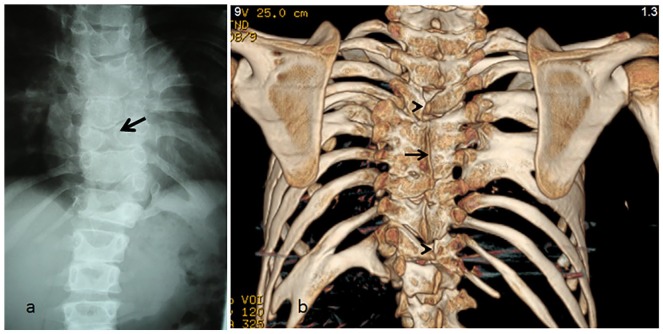
Loss of spine biomechanics. Anteroposterior spine radiograph in a 7-year-old child showed vertebral malsegmentation, that is, hemivertebrae and butterfly vertebrae (T6-11), respectively (arrow) (a) Three-dimensional (3D) reconstruction computed tomography (CT) scan of the spine and the thoracic cage in a 9-year-old boy with autosomal recessive Robinow syndrome presented with rigid spine movements (loss of the physiological spine biomechanics). He showed massive spondylothoracic malsegmentation associated with rib fusions and bifid ribs seen adjacent to the malsegmented spine segments. Strikingly, spina bifida occulta at the level of T3 and T10 (arrowheads) associated with a posterior malsegmented bar runs along T5-T8 on top of bilateral failure of segmentation. In addition to multilevel ribs fusions (right side, fusions of ribs 4 and 5, followed by fusions of 6, 7, and 8 ribs, respectively [arrow]). On the left side, fusions of ribs 6 and 7 followed by fusions of ribs 10 and 11 causing effectively the development of bifid rib (b).

**Figure 3.**
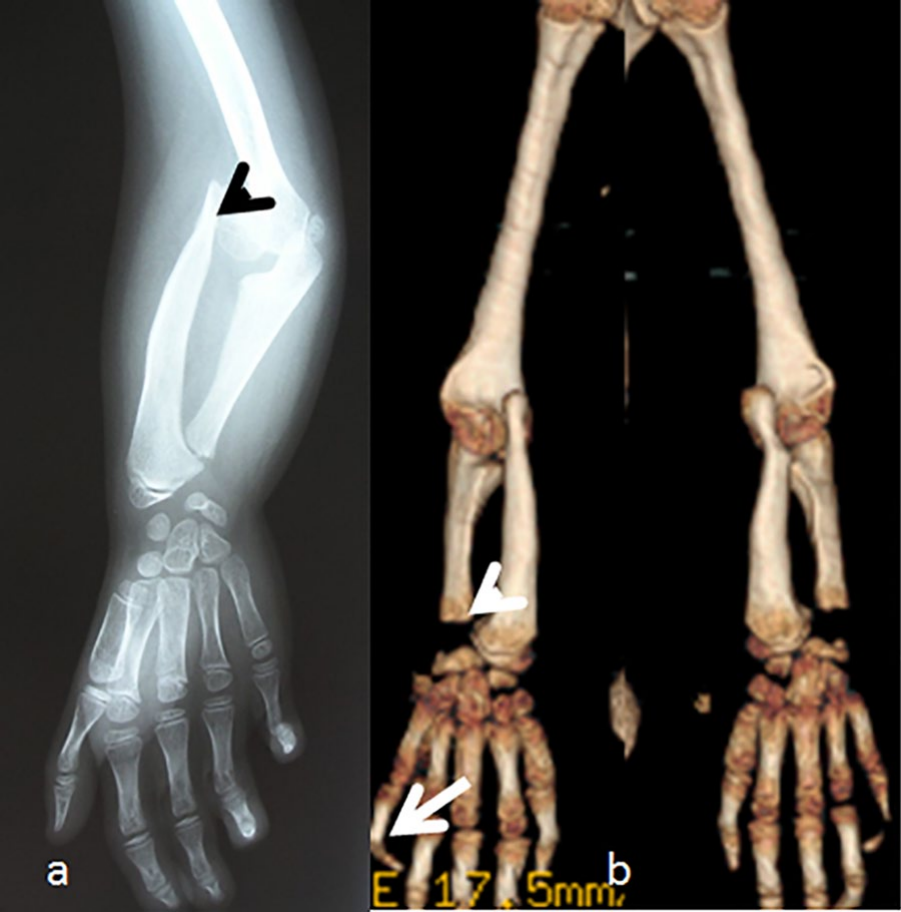
Radioulnar dislocation and severe retardation of the fine motor skills. Anteroposterior radiograph of the upper limb in a 7-year-old child. Note the marked mesomelia and the superior and inferior radioulnar dislocations (arrow head) with Madelung’s-like deformity associated with camptodactyly of the fourth and fifth fingers, respectively (a). Three-dimensional (3D) reconstruction computed tomography (CT) scan of the upper limbs in a 5-year-old girl with autosomal recessive Robinow syndrome showed bilateral radioulnar superior and inferior dislocation (Madelung’s deformity—arrow head) with marked dysplastic process over the epi metaphyseal components, small hands with bilateral camptodactyly of the fourth and fifth fingers (arrow). All patients with autosomal recessive Robinow syndrome presented with marked limitations in pronation and supinations because of mesomelic dysplasia secondary to Madelung’s deformity (b).

1. *Stature*: Growth deficiency of −4SD.
2. *Developmental history and intelligence*: All manifested subsequent developmental retardation and borderline intelligence with poor schooling achievements.
3. In this group, broad spectrum of clinical presentations such as intellectual disability, persistent headache, and episodes of dizziness have been recorded. Craniofacial contour has been analyzed via a 3-dimensional (3D) reconstruction computed tomography (CT) scan. A 7-year-old girl with AR type of RS complained of poor schooling achievements and persistent headache originating from the back of the skull. Three-dimensional reconstruction CT scan showed early closure of the metopic suture (arrowhead), associated with profound bossing of the frontal area (more marked at the glabella) notable features. Note the normality of the coronal sutures and acute depression of the nasofrontal angle with dysplastic supraorbital ridge (Figure 1a). Three-dimensional reconstruction CT scan in the same girl with ARRS showed early closure of the squamosal sutures (arrowhead; Figure 1b). Three-dimensional reconstruction CT scan in the same girl with ARRS showed normal lambdoid sutures with no trace of Wormian bones (Figure 1c).

4. A 7-year-old child showed vertebral malsegmentation, that is, hemivertebrae and butterfly vertebrae (T6-11), respectively (Figure 2a). A 9-year-old boy with RSS presented with rigid spine movements (loss of the physiological spine biomechanics) in addition to a history of recurrent bronchitis in his early life and shortness of breath during school sport lessons. Three-dimensional reconstruction CT scan of the spine and the thoracic cage showed massive spondylothoracic malsegmentation associated with rib fusions and bifid ribs seen adjacent to the malsegmented spine segments. Strikingly, spina bifida occulta at the level of T3/10 (arrowheads) associated with a posterior malsegmented bar runs along T5-8 on top of bilateral failure of segmentation. In addition to multilevel ribs fusions (right side, fusions of ribs 4 and 5, followed by fusions of 6, 7, and 8 ribs, respectively [arrow]). On the left side, fusions of ribs 6 and 7 followed by fusions of ribs 10 and 11 causing effectively the development of bifid rib (Figure 2b).

5. A 7-year-old child. Note the marked mesomelia and the superior and inferior radioulnar dislocations with Madelung’s-like deformity associated with camptodactyly of the fourth and fifth fingers, respectively (Figure 3a). A 5-year-old girl with RSS showed bilateral radioulnar superior and inferior dislocation (Madelung’s deformity) with marked dysplastic process over the epi metaphyseal components, small hands with bilateral camptodactyly of the fourth and fifth fingers. All patients with ARRS presented with marked limitations in pronation and supinations because of mesomelic dysplasia secondary to Madelung’s deformity (Figure 3b).

### Group II: Autosomal Dominant Pattern of Inheritance (DRS1) That Is Caused by Heterozygous Mutation in the WNT5A Gene on Chromosome 3p14

**Figure 4.**
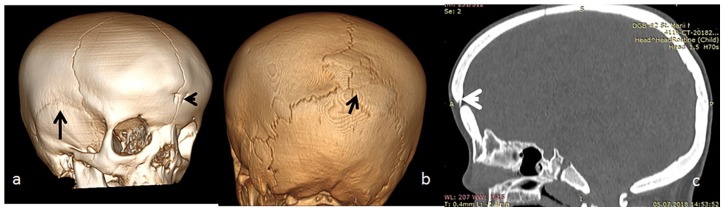
Normal cranium but presence of acute depression of the nasofrontal area and Wormian bones. Three-dimensional (3D) reconstruction of the cranium in a 7-year-old boy with Robinow syndrome, autosomal dominant type, showed persistence of the metopic suture associated with the development of bilateral dimples on the sides of the metopic suture (arrowhead). The squamosal sutures were normal (arrow). The acute depression of the nasal-frontal area is less marked than seen in the autosomal recessive type, similarly a less bulging/bossing of the frontal area. (b) Wormian bones along the lambdoid sutures were notable (arrow). (c) Sagittal 3D-reformatted computed tomography scan showed defective ossification of the frontal bone, which is correlated to the dimples along the metopic suture.

**Figure 5.**
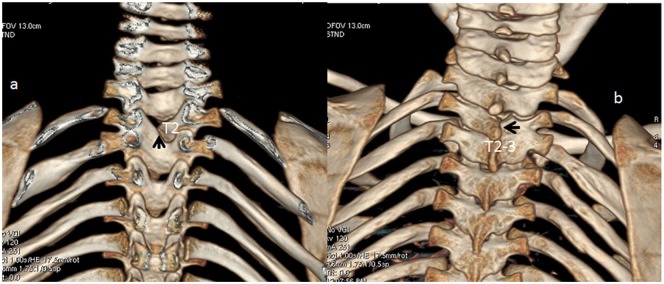
Thoracic kyphosis. Three-dimensional (3D) reconstruction computed tomography (CT) scan in a 6-year-old boy with autosomal dominant Robinow syndrome who presented with thoracic kyphosis showed malsegmentation of T2/3 (anterior view-arrow). The 3D reconstruction CT scan (posterior view) confirmed the malsegmentation along the T2/3 and not associated with rib fusions.

**Figure 6.**
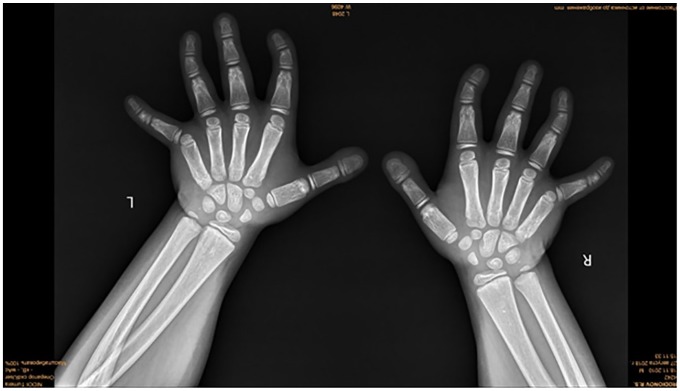
Neither Madelung’s deformity nor radioulnar dislocation. Anteroposterior radiograph of the forearms in a 5-year-old boy with Robinow syndrome, autosomal dominant type, showed normal development of the radioulnar with normal pronation and supination. No camptodactyly, though rectangular first metacarpals, multiple pseudoepiphyses, and clinodactyly, were noted.

1. *Stature*: Growth deficiency of −3SD.
2. *Developmental history and intelligence*: All manifested subsequent developmental retardation but normal intelligence.
3. *Craniofacial*: Three-dimensional reconstruction showed persistence of the metopic suture associated with the development of bilateral dimples on the sides of the metopic suture. The squamosal sutures were normal. The acute depression of the nasal-frontal area is less marked than seen in the AD type, similarly, less bulging/bossing of the frontal area (Figure 4a). Wormian bones along the lambdoid sutures were notable (Figure 4b). Sagittal 3D-reformatted CT scan showed defective ossification of the frontal bone, which is correlated to the dimples on the sides of the metopic suture (Figure 4c).

4. *Spine*: In all patients, vertebral malsegmentation was the most notable features, not associated with rib fusions and or unsegmented bars. A 6-year-old boy with ADRS presented with thoracic kyphosis. Three-dimensional reconstruction CT scan, anteriorly (a) showed malsegmentation along T2/3 and posterior view (b) clearly showed the fusion of T2/3 without rib fusions (Figure 5a and b).

5. *Upper limbs*: No mesomelic dysplasia has been elicited in these patients. Radiographs showed normal development of the radioulnar with normal pronation and supination. Anteroposterior radiograph of the forearms in a 5-year-old boy with ADRS showed normal development of the radioulnar with normal pronation and supination. No camptodactyly, though rectangular first metacarpals, multiple pseudoepiphyses, and clinodactyly, were noted (Figure 6; refer to Tables 1 and 2).

**Table 1.** Descriptive Phenotype/Genotype With Autosomal Recessive and Autosomal Dominant Types of Robinow Syndrome.

Age	Sex	Growth	Developmental Skills and Complaint	Phenotype/Genotype
7 years	Female	−4SD	Retarded motor and fine motor, intellectual disability, and episodes of migraine	ARRS/homozygous mutations in the *ROR2* gene on chromosome 9q22
6 years	Female	−4SD	Retarded motor and fine motor, dislocated radioulnar, and intellectual disability	ARRS/homozygous mutations in the *ROR2* gene on chromosome 9q22
5 years	Male	−4SD	Retarded motor fine motor and loss of spine biomechanics	ARRS/homozygous mutations in the *ROR2* gene on chromosome 9q22
7 years	Male	−3SD	Retarded motor but normal fine motor and normal intelligence	ADRS/heterozygous mutation in the WNT5A gene on chromosome 3p14
5 years	Female	−3SD	Retarded motor and thoracic scoliosis	ADRS/heterozygous mutation in the *WNT5A* gene on chromosome 3p14 heterozygous mutation in the *WNT5A* gene on chromosome 3p14
5 years Related 9 years (cousin of the 5-year-old boy)	Male Female	−3SD −3SD	Retarded motor, spina bifida occulta of the cervical spine Thoracolumbar scoliosis	ADRS/heterozygous mutation in the *WNT5A* gene on chromosome 3p14 No genetic tests (cousin of the 5-year-old boy)

Abbreviation: ARRS, autosomal recessive pattern of inheritance; ADRS, autosomal dominant pattern of inheritance.

**Table 2.** Complexities of Genetics in Robinow Syndrome.

Robinow Syndrome	Defective Genes
Autosomal recessive pattern of inheritance	*ROR2, WNT5A*, and *NXN*
Autosomal dominant pattern of inheritance	*WNT5A, Fzd2, RYK, DVL1*, and *DVL3*

## Discussion

The human skull is composed of several bones that fuse together after birth. The bones of the skull can be divided into the viscerocranium and the neurocranium. The viscerocranium consists of the bones that make up the bones of the face and the pharyngeal arches. The neurocranium consists of the bones protecting the brain and sensory organs.^38^ The calvarium of the skull consists primarily of large flat bones separated by bony sutures. Craniosynostosis is a condition characterized by premature fusion of cranial sutures. The sutures generally fuse at the end of the second year of life. Previous studies described the extent of complications that might ensue in correlation with early fusion of the cranial sutures.^39^

Surgical correction of craniosynostosis has to be done early in the child’s life. The management of abnormal craniofacial anatomy, specifically the correction of craniosynostosis, is recommended between 3 and 6 months of age.^40^

In our group of patients with autosomal recessive type of Robinow syndrome, it seems extremely late to intervene surgically in order to repair the synostotic sutures. Vertebral anomalies occurring during the mesenchymal stage may be due to either a unilateral defect of formation or segmentation of the primitive vertebrae and can result in a unilateral imbalance in the longitudinal growth of the spine-producing congenital scoliosis.^41^ Al Kaissi et al^42^ described a 13-year-old girl presented with spondylocarpotarsal synostosis syndrome associated with a midline unsegmented bar extending over the posterior spinal processes. Untreated progressive early-onset spinal deformities can lead to a series of morbid complications such as respiratory insufficiency and pulmonary and cardiac hypertension, which characterize thoracic insufficiency syndrome, which might be fatal. Thoracic insufficiency syndrome is the inability of the thorax to ensure normal breathing. This clinical condition can be linked to variable axial anatomical disruptions such as fused ribs, hemivertebrae, and congenital unsegmented bars.

The only article that described the craniofacial and intraoral phenotype of Robinow forms was the study by Beiraghi et al.^43^ They concluded that there were differences in the severity of the craniofacial and intraoral features between the autosomal dominant and the recessive forms of RS. But, nevertheless, their findings did not include distinctive study of the skull bones and its correlation with the intraoral anatomy.

## Conclusion

Tomographic studies are useful tools used to detect the pathological mechanism and to further understand the reasons behind the frequent clinical complaints in children/adults with syndromic malformation complex. Congenital osseous disruptions can hardly get interpreted via conventional radiographs, simply because of the anatomical overlap of the malformed osteogenic structures. To differentiate between the 2 types of RS and to further interpret the reasons behind the clinical ailments and the frequent complaints in these children, we referred to 3D reconstruction CT scan. We believe that tomographic studies could replace the high cost of molecular and genetic tests in RS patients, and it could alleviate the burden for the patients and their families.

Previous reports emphasized solely on the clinical management of orofacial pathologies in patients with RS. Scrutinizing the literature revealed insufficient explanation regarding the etiology behind the abnormal craniofacial contour, axial and appendicular malformations, and the correlated symptomatology. Early closure of the metopic suture and anterior part of the sagittal and the squamosal sutures requires special attention and early surgical interventions to overcome the intellectual disability and to lessen the episodes of migraine in the ARRS type. Similar plans are required to lessen the complications of axial and appendicular osteogenic deformities in the ARRS type. The connection between the broad-spectrum clinical presentations in RS with existing anatomical malformation complex has never been described in the literature. Three-dimensional reconstruction CT scan is the modality of choice for the precise assessment of the malformed skeletal system.
